# Supplementary material for: Are carbon emissions trading and green financial instruments synergistic? -Comprehensive quantitative research based on content analysis
Source: PLoS One. 2024 Mar 7;19(3):e0298601. doi: 10.1371/journal.pone.0298601 (PMC10919601; doi:10.1371/journal.pone.0298601)
Supplement: S1 Data — (ZIP) [file pone.0298601.s001.zip › Data/地级市Z_F工作报告.zip!/║┌┴·╜¡/─╡╡ñ╜¡╩╨/└┤╫╘╣1⁄2╓┌║┼í╛┬φ┐╦╩2╛▌═°í┐_2016.docx]

各位代表：

　　我代表市人民政府，向大会报告工作，请予审议，并请市政协委员提出意见。

　　一、2015年工作情况和“十二五”时期简要回顾

　　过去的一年，在省委省政府和市委的正确领导下，在市人大、市政协的监督支持下，我们紧紧依靠全市人民，积极应对经济下行压力， 按照市委“三步走”目标， 强力推进“九增行动”，经济社会稳步发展，“十二五”圆满收官。 地区生产总值实现1318.4亿元、 增长6.6%； 固定资产投资完成1019.9亿元、 增长10.5%；规上工业增加值实现270.9亿元、增长4.5%；社会消费品零售总额实现526.8亿元、增长10.8%；进出口总额实现48.9亿美元，总量保持全省第一方阵；一般公共预算收入完成59亿元,税收占比达到65.5%、高于同期16.5个百分点；城镇常住居民人均可支配收入达到26673元、增长7.8%，农民人均纯收入达到18535元、增长10%。 固定资产投资、社会消费品零售总额增速列全省第一，地区生产总值、规上工业增加值增速列全省第三。

　　一是争取国家政策和重大项目实现新突破。主动对接国家战略、重大项目和重点改革，我市成为国家生态文明先行示范区、全国区域级流通节点城市，市经开区获批国家循环化改造示范试点园区，温春镇获批国家建制镇示范试点； 全市对上争取资金165亿元、增长7.4%；重大项目开复工顺利，荒沟抽水蓄能电站、奋斗水库建设取得新进展，哈牡客专全线开工，牡绥扩能改造建成运营。

　　二是产业项目建设取得新进展。 开复工500万元以上产业项目1433个，完成投资623亿元、增长29.5%，产业投资比重达到61.1%； 新开工产业项目1250个、同比增加601个、完成投资537.1亿元，数量、增幅均居全省第1位。新开工太敬机器人等亿元以上工业项目29个、百威英博等10亿元以上工业项目4个，工业投资增长21.8%；外资实际到位4.6亿美元、增长2.8%。园区建设和招商引资有新成效，市经开区开复工亿元以上项目73个，宁安、东宁、爱民、西安、阳明、东安6个县（市）区为市经开区引进项目取得实质进展， 宁安用市场机制推进园区土地收储、基础设施和项目建设，得到省政府肯定； 全市签约亿元以上项目115个，引进域外资金736.6亿元、增长17.2%。工业稳步增长，新增规上企业23户，首控石油等46户规上企业恢复生产；桦林佳通等3户企业获批大用户直供电； 友搏药业借壳主板上市，天合石油、华安新材在“新三板”挂牌，富通空调与上市公司资本重组借道发展，我市结束近15年没有企业上市的历史；落实省“千户科技型企业三年行动计划”，新形成销售收入500万元以上科技型企业63户，新增国家级高新技术企业9户，省级以上企业研发机构、省级科技企业孵化器分别达到41家和10家，高新技术产业增加值增长15.3%。

　　三是特色精品农业发展再上新台阶。综合生产能力持续提升，粮食总产达到66亿斤，再创历史新高。特色农业加快发展， 以食用菌、棚室蔬菜、畜牧业、林下经济为主的特色产业， 占农村经济总收入比重达到48.6%。产业化、市场化步伐加快， 省级以上龙头企业和农民专业合作社发展到46户和1980个，绿色有机食品认证面积达到455万亩，驰名著名商标和国家地理标志产品发展到33个和5个，宁安响水大米、东宁黑木耳被评为全国品牌价值超50亿元的地理标志产品。

　　四是现代服务业赢得新提升。旅游经济高速增长，新增3A级以上景区4个，雪乡景区收入大幅增长,全市接待游客1483.3万人次、实现旅游收入93.1亿元，分别增长26%和35.1%，民航客运量和出入境客运量突破65万和10万人次，分别增长19.8%和13.3%。市经开区成为国家电子商务示范基地，海林被确定为全国电子商务进农村示范市。金融服务业取得新进展，新增贷款余额80.7亿元、增长13.4%，金融产业增加值增长10.5%。文化产业健康发展，中俄文化艺术品交易中心网络平台投入运营，文化产业增加值实现38亿元、增长15%。

　　五是对外合作迈出新步伐。 落实市委“发展开放型经济、建设开放型城市”主导战略。7个境外园区入驻企业发展到80户，境外农业合作面积达到480万亩、回运粮食6.3万吨，开通“哈牡绥俄亚”专列，俄海产品进口实现零突破；交通枢纽国际物流中心一期投入使用，华晟物流二期保税监管仓库竣工，绥芬河全国首个中俄跨境电商监管中心“中俄云仓”启动运营， 全市集装箱跨境物流货运量突破30万吨、增长46%。全市跨境人民币结算量58.5亿元、居全省地市首位，绥芬河卢布现钞使用试点正式启动。

　　六是城乡建设展现新风貌。坚持规划先行，编制完成牡丹江风貌特色规划等14个专项规划。城市交通路网格局有新变化，新安街西延工程全线通车，南北太平路贯通工程、牡丹江大桥拓宽工程开工建设，整修西七条路等6条道路； 推进文明城市创建“十大工程”和城市管理提升工程，市区全面实施绿色清雪、机械化水平达到70%以上， 新建和改造供热、燃气管网30公里。认真办理市人大“一号议案”，市区实现物业管理全覆盖。推进国家新型城镇化综合试点建设，牡海和海长公路通车为雪乡旅游和沿线发展提供了便利条件，东宁撤县设市获批， 穆棱下城子镇荣获全国文明镇、全省百强镇第一名； 开复工与森工、农垦共建项目50个、完成投资9.6亿元。户籍人口城镇化率达到56.6%。 加快美丽乡村建设， 开展农村环境整治百日会战行动，新建改造农村公路308.4公里， 新增农村饮水安全村屯125个、惠及9万人，建设美丽乡村示范村41个、达标村139个。

　　七是民生事业获得新发展。 20件利民实事全部完成年度任务，民生支出132亿元、占一般公共预算支出54.7%。全面启动“大众创业、万众创新”，推动创业带动就业，发放小额担保贷款1.1亿元，城镇新增就业6.2万人，登记失业率控制在3.38%，我市农民工工作获国务院表彰。社会保障扩面提标，在全省率先实行城镇居民大病保险市级统筹，五项社保新增参保2.9万人； 企业退休人员和城乡居民基础养老金标准分别提高12.3%和27.3%， 城乡低保标准分别提高13.6%和29.6%，农村五保供养标准提高20%。人居环境持续改善，完成棚户区改造1.1万套，改造农村泥草（危）房1.5万户。社会事业全面发展，全市义务教育学校标准化率达到91.2%； 县级公立医院改革全面启动，公办基层医疗卫生机构全部配备使用国家基本药物并实施零差率销售；深入开展文化体育惠民，全市建设中心村文化中心广场158个，《林海雪原》、《八女投江》等8部影视作品在我市拍摄； 改善城乡生态环境，取缔改造燃煤锅炉124台，淘汰黄标车8500辆，市污水处理厂二期和生活垃圾处理厂二期投入使用； 新建城乡达标社区49个， 城市社区居家养老服务和“儿童之家”实现全覆盖；社会保持平安稳定，深入开展食品药品、安全生产专项整治，启动专业化反恐处突巡防体系建设，全市安全生产事故起数、 死亡人数分别下降16%和55.3%，刑事案件、治安案件分别下降7.4%和12.5%，“平安牡丹江”官方微信公众平台获全国政务头条号“特别贡献奖”；实施联合接访、领导包案制度，解决疑难信访问题169件， 解决历史遗留的“一房多售”2000户、“动迁未回迁”1万户， 全市集体访、进京访分别下降18%和36.8%。

　　八是政府自身建设取得新进步。深化行政审批制度改革，集成职能、再造流程、简化审批、限时办结，企业注册登记办理时限压缩至1小时内、现场办结率100%，全市新增市场主体2.1万户、增长75%。 统筹推进政府机构和事业单位分类改革，完成市、县（市）区机构改革，撤并转事业单位机构17个；成立城市地下管线综合服务中心，消除地下管网“九龙治水”乱象。加快法治政府建设，推进综合执法体制改革。持续优化发展环境， 建立政府权力清单、责任清单；7家“红顶”中介与主管部门脱钩，与审批有关的市属中介收费下调50%；取消和免征各项收费1.4亿元；强化企业投诉中心功能，实施涉企“宁静工程”和“门禁制度”，查办影响发展环境问题42件。深入开展“三严三实”专题教育，加强勤政俭政廉政建设，完善市政府工作规则及制度，强化政策落实、财政预算执行、税收征管和建设项目投资审计，“三公”经费支出缩减5%。办理人大代表议案3件，建议、批评和意见215件，办理政协提案298件，均得到有效落实和答复。

　　统计、气象、地震、档案、公积金、人防、边防、民兵预备役、民族宗教、妇女儿童、残疾人、红十字等工作都取得了新成绩，驻牡中省直部门、驻牡部队为地方发展做出了突出贡献。

　　各位代表，“十二五”时期，在市委的正确领导下，我们认真贯彻落实省委省政府的战略部署，全市各项事业取得重大进展。

　　——五年来，我们综合施策稳增长促发展，综合实力显著增强。 地区生产总值年均增长11.2%，经济总量跃居全省第3位；一般公共预算收入年均增长6.6%， 人均财力是2010年的1.9倍； 固定资产投资四年翻一番；社会消费品零售总额五年翻一番。绥芬河、海林、穆棱、宁安保持全省十强，林口进入全省工业发展十强。先后争取到国家老工业区搬迁改造试点、国家新型城镇化综合试点地区、国家跨境贸易电子商务服务试点、国家新能源示范城市、全国水生态文明城市建设试点等国家级试点。

　　——五年来，我们强力推进产业项目建设，结构调整取得积极进展。引进产业投资1609.8亿元，是“十一五”时期的3.7倍，其中工业投资643.9亿元，是“十一五”时期的3.8倍，建成亿元以上产业项目133个，国家级开发区发展到2个。 特色农业加快发展，“菌菜牧林瓜”等优势产业初步形成，市级以上龙头企业发展到195户，成为中国绿色有机食品之都、世界黑木耳之都。工业经济稳步提升， 规上工业增加值五年翻一番。现代服务业规模扩张，旅游收入四年翻一番。

　　——五年来，我们着力推动对俄合作升级，沿边开放进入新阶段。境外园区发展到7个，其中3个晋升为国家级，建成全省唯一综合保税区。华晟国际物流实现口岸功能内移，跨境陆海联运通道打开了龙江的出海口。我市成为全国中俄地区友好合作示范城市，有经贸往来的国家和地区达到136个。

　　——五年来，我们统筹推进城乡建设，城乡面貌发生明显变化。深入开展文明城市、美丽乡村和新型城镇化试点建设。牡三公路等16条公路和兴隆大桥等9座桥梁相继通车， 新建11条、翻建91条城市道路，推进“三溪一河”治理、十大主题公园等一批惠民工程，城市框架和功能进一步提升。开展与森工、农垦合作，局市共建项目151个、完成投资40.1亿元，全市转移农业人口8.9万人。提前完成“十二五”减排任务，治理水土流失180万亩，我市成为全国绿化模范单位。

　　——五年来，我们切实保障改善民生，人民群众生活水平不断提高。坚持每年办成一批利民实事，民生支出总额是“十一五”时期的2.5倍。农民人均纯收入实现全省“十三连冠”。建设保障性住房1.5万套，改造农村泥草（危）房6.5万户，15万城乡居民住房条件明显改善。五项社会保险扩面30万人、标准全部提高,企业退休人员养老金实现“十一连增”；城乡低保标准分别增长74.8%和1.7倍，累计发放救济救助资金超过12亿元。 社会事业加快发展，完成168所学校、8所医院标准化建设， 我市成为国家公共文化服务体系示范区。食药、生产、治安等公共安全保障全面提升，社会保持和谐稳定。

　　——五年来，我们坚定不移深化政府改革，发展活力和动力逐步增强。推进服务型政府建设，精简行政权力事项69.5%，开通网上审批中心， 实施商事登记“一表制”、建设项目审批“一图三会制”，新增市场主体五年增长1.3倍。 赋予市经开区市级管理权限。推进城市管理“一队式”综合执法，提高了政府管理水平。持之以恒抓廉政、转作风、强服务，经济发展环境不断优化。

　　各位代表，过去五年取得的成绩，得益于省委省政府和市委的正确领导，得益于市人大、市政协的有力监督和全力支持，得益于全市人民团结一致、不懈拼搏，得益于各驻牡单位的支持和帮助，得益于历届政府奠定的坚实基础。 在此，我代表市人民政府，向全市人民、人大代表、政协委员， 向离退休老领导、老同志，向各民主党派、工商联、人民团体，向各中省直单位，向驻牡部队和武警官兵，向所有关心、支持牡丹江发展的各界人士和国际友人，致以崇高的敬意和衷心的感谢！

　　我们清醒地认识到，我市发展还存在不容忽视的问题：经济下行压力较大，产业规模不大、结构不优、竞争力不强、科技创新根基不牢、财政保障能力不强等问题比较突出；在就业社保、公共服务、社会治理、生态环境等方面与百姓期盼还有较大差距；深层次的思想理念落后、体制机制不活、市场化程度不高、发展环境不优等问题还需要下大功夫解决；政府部门在法治化、专业化、先进管理方式和手段应用、勤政俭政廉政建设上还需进一步努力。对此，我们将直面矛盾、正视问题、全力作为，在今后的发展中认真加以解决。

　　二、“十三五”奋斗目标和主要任务

　　“十三五”时期，是全面建成小康社会的决胜阶段。总体看,加快发展机遇与挑战并存,机遇大于挑战。

　　从挑战看，除了我市自身存在的问题外，国际上，世界经济复苏乏力， 国际贸易低迷徘徊，以TPP为代表的国际贸易规则重塑，地缘政治冲突加剧，使外部经济环境复杂多变，汇率的大幅波动，出口的针对性壁垒，对我市外向型经济形成冲击；我国经济“三期”叠加的阶段性影响还在释放，经济发展新常态下，速度变化、结构优化、动力转换三大特点，倒逼我市加快转方式、调结构、抓创新；在省内地市竞相发展的态势下，农业规模小、矿产资源少、工业缺支柱、现代服务业缺人才和科技支撑、区位处于国界省边，在产业项目战略布局选择和资源重大项目生成中存在不利因素。

　　从机遇看， 国际上，俄罗斯海参崴自由港政策、“滨海1号”大通道建设、中韩自贸协定生效等，给我市带来千载难逢的开放机遇； 我国全面深化改革、“一带一路”战略、 新一轮东北振兴、大众创业万众创新、加强生态文明建设等政策红利，给我市带来重大发展支撑；我省实施量身定制的国家“五大规划”，中蒙俄经济走廊项下的“龙江丝路带”建设，加快十大重点产业发展等，给我市精准施策提供了方向引领；我市4个一类口岸的开放条件、生态自然条件、 公铁枢纽城市的区位功能优势在“十三五”的强化，加之全市团结奋进、真抓实干、风清气正的政治生态，将为加快发展凝聚增长新动能，正是天时、地利、人和。我们要科学研判、精准发力、抢抓机遇、应对挑战，不断抢占新一轮战略布局和经济竞争的制高点。

　　“十三五”时期全市经济社会发展的总体要求是：全面贯彻落实党的十八大和十八届三中、四中、五中全会以及中央经济工作会议精神，深入贯彻习近平总书记系列重要讲话精神，按照“五位一体”总体布局和“四个全面”战略布局，牢固树立和贯彻落实创新、协调、绿色、开放、共享的发展理念，积极适应经济发展新常态，坚决落实省委省政府和市委各项部署，坚持把发展作为第一要务，把创新作为第一动力，把提高经济发展质量和效益作为中心任务，创新实施“五大规划”发展战略，深入推进“龙江丝路带”建设，紧跟省十大重点产业发展步伐，以发展开放型经济、建设开放型城市为主攻方向，以全面改善民生为根本目的，加快现代化、国际化城市建设，确保如期实现全面建成小康社会奋斗目标。

　　“十三五”时期发展的总体目标是：实现地区生产总值比2010年提前一年翻一番，城乡居民人均收入稳定实现提前两年翻一番，全市脱贫任务提前三年完成。在此基础上，努力实现更好更快发展，力争地区生产总值与城乡居民人均收入年均同步增长7%左右，城市综合竞争力明显提升。

　　“十三五”时期发展的主要任务：

　　（一）坚持创新发展，提升经济实力。加快培育现代产业体系，着力发展林木林纸、绿色食品、精细化工、装备制造、新型建材、生物医药、现代旅游、现代物流8个重点产业， 积极发展新材料、新能源、机器人、信息产业4个新兴产业，力争打造医药、食品、装备、林木林纸、旅游5个百亿级产业；更加注重一二三产业融合发展，优化“粮经饲药菌”结构，实现境内、境外、林下“三个千万亩”，走生态绿色有机、特色高效精品农业发展道路。激发创新活力，深入实施创新驱动战略，大力推进“大众创业、万众创新”，积极落实省“千户科技型企业三年行动计划”，2020年初步建成创新要素集聚、创业体系完备、政策环境优越的创新型城市。 创新发展体制机制，推动政府信息化、网络化、集成化、后台化改革， 构建政府、市场、社会、国际综合公共服务平台，在产业集聚、创新驱动、人才吸引、要素整合、市场活跃上，构建优良服务环境，成为面向东北亚的服务高地、成本洼地、产业集聚地。

　　（二）坚持协调发展，增强载体功能。提升中心城市品质品位，打造“牡海宁”中心城市圈，高标准、高质量建设江南新城，五年内中心城市建成区面积和户籍人口分别达到90平方公里和90万人以上。加快推进国家新型城镇化综合试点， 全市户籍人口城镇化率达到65%以上；六县（市）全部进入全省十强县，四城区进入全省城区第一方阵，力争市经开区综合实力进入省内开发区前五位。按照全省“六美”目标，建成美丽乡村示范村100个、达标村400个，美丽乡村建设走在全省前列。坚持物质文明精神文明同步提升，加强思想道德建设，培育城市人文精神，繁荣文化产业和事业，活跃城乡文化市场，支持牡丹江特色文化精品创作，创成全国文明城市。促进军民融合发展，在机器人、卫星应用、网络通信、大数据等领域成为国家军民融合产业示范。

　　（三）坚持绿色发展，凸显生态优势。倡导绿色生活方式，养成尊重自然、保护生态、敬畏山水、热爱动植物的文化风尚，促进人与自然和谐相处。发展生态产业，突出抓好以镜泊湖生态恢复性开发为重点，以生态旅游、养生养老、医疗美容、休闲娱乐等业态为主线的生态产业，保护性开发利用好三道关、牡丹峰等9个国家森林公园，保护好火山口、牡丹峰2个国家自然保护区和宁安白桦川、东宁绥芬河2个国家湿地公园,争取镜泊湖世界地质公园等列入国家公园，建成牡丹江沿江、海浪河等一批国家湿地公园，建设环城山群各具特色的森林休闲公园。优化生态环境，深入开展大气、水域、土壤污染防治，完成林海水库建设，推进饮用水源地综合整治和海浪河源头生态保护， 着力解决关系人民群众健康的环境污染问题，2020年单位GDP能耗、水耗和建设用地占用面积全部达标,市区空气质量优良天数达到80%以上。加大退耕还林还湿还草力度，实施以“水气山林绿”为重点的生态保护修复和生活环境改善工程，2020年森林覆盖率达到65%以上， 牡丹江流域水质优良比例达到75%以上。创建国家森林城市和国家园林城市。

　　（四）坚持开放发展，放大区位优势。提升城市开放功能，加速推进基础设施互联互通，加快铁路、公路、机场、口岸和管网建设，重点建设“七铁两路一桥两机场”的开放大通道，成为东北东部高铁和高速公路节点城市、航空枢纽城市。加快开放型经济建设，构建开放型产业体系，2020年全市争取培育进出口额超亿美元外向型企业10户以上，进口资源落地加工率达到60%以上，地产品出口比重达到20%以上。加速推进开放平台建设，推进对俄跨境电商平台，构建牡丹江国家综合保税区、绥芬河国家综合保税区、绥东重点开发开放试验区、穆棱边境经济合作区为龙头， 境内外园区为支撑的对外开放平台体系。 深化科技、文化、教育、卫生、旅游、体育等领域对外交流合作。

　　（五）坚持共享发展，增进群众福祉。按照市委执政为民、施政利民、从政亲民、察政问民的要求，坚守底线、突出重点、引导预期，注重机会公平，保障基本民生。从解决人民群众最关心、最直接、最现实的利益入手， 全面落实学有所教、劳有所得、病有所医、老有所养、住有所居的各项任务， 更加注重食品安全、生产安全和社会平安，提升义务教育、就业服务、社会保障、基本医疗和公共卫生、公共文化、环境保障等基本公共服务的实现能力和水平。坚决打赢脱贫攻坚战，2017年实现农村贫困人口全面脱贫，确保一个都不少的与全市人民同步实现小康。 突出抓好保障性安居工程建设， 2020年前全部完成城镇棚户区、城中村、农村泥草（危）房改造任务。强力推进依法治市，创新社会治理模式，2020年基本建成法治政府。

　　三、2016年重点工作任务

　　　　2016年是全面实施“十三五”规划的起步之年，是全面建成小康社会决胜阶段的开局之年。我们将按照市委十一届九次全会工作部署，深入贯彻落实省委十一届六次全会和省委经济工作会议精神，以落实“五大发展理念”为主线，以发展开放型经济、建设开放型城市为主攻方向，坚持稳中求进总基调，着力加强结构性改革，去产能、去库存、去杠杆、降成本、补短板，提高供给体系质量和效率，提高投资有效性，加快培育新的发展动能， 改造提升传统比较优势， 增强持续增长动力，全力抓好稳增长、调结构、惠民生、防风险，加快经济发展，保持社会和谐稳定，确保“十三五”我市经济社会发展良好开局。

　　经济社会发展预期目标是： 地区生产总值增长7%左右，固定资产投资增长10%以上，规上工业增加值增长7%以上，社会消费品零售总额增长8%以上，城乡居民人均收入均增长7%，一般公共预算收入和外贸进出口总额均增长3%以上，万元GDP综合能耗下降3.5%，经济社会发展整体水平保持全省前列。

　　重点抓好九个方面工作：

　　（一）全力争取国家和省重大支持。抓住国家、省编制实施“十三五”规划纲要和各专项规划的战略机遇，以及各项重大政策出台的关键节点，积极对口对接，全力争取重大政策、重大项目、重大开发开放平台和重大改革试点，在基础设施、战略性新兴产业、公共服务、对外开放、生态治理、政府改革等方面，争取国家和省更多支持。加快投资354亿元的哈牡客专建设，力争开工建设投资387亿元的牡佳客专、46.5亿元的哈牡既有线电气化改造等重大项目，确保高寒城市智能公交系统、荒沟抽水蓄能电站、小莲花水电站、奋斗水库等项目完成年度投资计划，力争牡丹江机场、绥芬河机场、 林海水库工程开工建设， 积极推动牡沈高铁、牡图电气化改造工作进程。

　　（二）强力推进产业项目建设。以供给侧结构性改革促进产业加速发展和结构调整。一是向存量释放要增量。巩固现有工业基础，加大规上企业服务力度，加速推进恒丰纸业、桦林佳通、富通空调、首控石油等龙头企业技术改造和产品创新，推动老企业搬迁改造、提档升级。二是向产业链延伸要增量。推动农林牧渔产品“接二连三”，在绿色食品、医药、生物大健康产业集群上整合资源包装引进大项目，创出更多全国著名品牌。在装备制造、新材料、新能源、对外贸易、化工等产业上进行补链招商引资，引进战略投资者加快兼并重组、整合上市，形成新的产业竞争优势。三是向战略性新兴产业和高新技术产业化要增量。按照省委要求，结合资源禀赋和产业基础，在航天航空、高端装备制造、新能源、新材料、生物医药、新一代信息技术等战略性新兴产业培育新的增长点。充分发挥我市卫星地面接收站、陆地光纤进出境端口等战略资源的独特优势，集合冷凉资源适中、电力资源富集等相对优势，在卫星遥测遥感数据开发应用、云计算及大数据数据中心建设和应用方面，与相关部委、科研机构、龙头企业合作，打造大数据加工及服务外包产业基地。在高端装备制造上，充分发挥与中科院、哈工大、哈工程等合作基础，形成机器人、医疗设备产业集群和产品集成； 引进战略合作者， 做强做大北方工具机器人、太敬机器人、低温等离子等龙头企业。在新能源产业上，推动投资33亿元的中诚通生物质能源基地建设，加快北方华仪风电装备、金旭韩华太阳能电池组件的市场推广，加快风场、地热资源的开发利用。在新材料产业上，结合我市矿产资源和现有企业，围绕石墨烯、碳化硼、玄武岩轻型材料等方向，引进战略投资企业。在生物医药产业上，支持友搏、黑宝、灵泰等一批企业发展，充分利用牡丹江生物多样性优势、企业药品批号资源和对俄对韩合作基础，吸引更多企业和项目集聚。 落实省“千户科技型企业三年行动计划”，内扶外引培育有规模科技型企业100户以上，新增孵化机构15家，高新技术产业增加值增长10%以上。 四是向大众创业、万众创新要增量。鼓励科技人员创新创业，吸引科技人员面向市场与企业结合、与资本结合；推动大学生创新创业，鼓励大学、科研院所兴办孵化器和创客空间，盘活大学周边厂房和办公楼为大学生提供创业空间，鼓励企业、农业经济组织与大学生合作；推动农民创业，促进农民创品牌、拓规模、闯市场、增资本。充分利用互联网整合资源、打通产业、协同创新、聚合要素、促进流通的作用，大力推动“互联网＋”行动计划，在农业、制造业、旅游、物流、传统商贸、金融、医疗、教育、传统媒体及文化产业、国际交流等方面加大应用和推广力度，催生新产业、新业态、新商业模式、新生产方式、新生活方式。 全市发放创业担保贷款1亿元以上，扶持创业2万人。 充分利用媒体宣传，烘托全市创业氛围，培植创业基因。五是向县域经济和非公经济发展要增量。做大做强做优县域经济是牡丹江经济发展的重要支撑。各县（市）要着力打造一个产业特色鲜明、税源拉动明显的集中园区，培育2—3个支柱产业。加大对非公经济的支持力度，落实“非公经济发展三年行动计划”和“中小微企业成长工程”，新增小微企业1000户以上。完成厂办大集体改革。六是向特色资源和区位要增量。积极对接省地质勘探三年专项行动，加快生成新矿。依托我市石墨、高岭土资源进行精深加工，力争石墨烯、特种陶瓷等产业的龙头企业落户。积极发挥区位优势，在俄矿、木、渔、油气深加工上形成产业集群。七是向产业集聚效应和园区载体服务要增量。深化市经开区体制改革，提升综合实力，力争牡丹江烟厂、珍宝岛药业等10亿元以上产业项目开复工5个以上，开复工龙穆黑牛等亿元以上项目30个以上。 提升各县（市）区园区载体功能，在电、上下水、燃气、蒸汽、热、通讯等基础保障上争取最低成本、最优服务。

　　要实现这些增量，有赖于精心地超前谋划、用心地招商引资、倾心地热情服务。要认真跟踪近两年参加我省组织的央企合作对接、民企龙江行、港台及国外招商活动形成的项目源，对七个“要增量”形成产业路线图和项目库，瞄准世界500强、产业50强、央企、民企100强，“定点爆破”精准招商，“按图索骥”抓产业链和产业集群，“踏石留印”落实服务责任；深入开展“四比”活动，全市力争开复工亿元以上产业项目100个左右，引进域外资金和实际利用外资分别增长15%和12%。

　　（三）致力加快特色精品农业提质增效。 特色精品农业是我市农民收入连续十三年领跑全省的 “金钥匙”。一是推动特色农业向高效精品农业转型升级。加快农业结构向“菌菜牧林瓜”等优势产业调整，促进农林牧渔相结合，推动种养加销一体化发展，全市新增经济作物15万亩，大榛子、红松坚果林、北药等新增10万亩，食用菌栽培规模达到43亿袋（块），畜牧业产值达到75亿元。二是突出新型主体、高端品牌、精深加工三个关键点， 提升农业产业化水平。 新增龙头企业10户，家庭农场、规范化农民专业合作社分别达到1150个和300个，新认证绿色有机食品品牌10个以上，进一步提高绿色有机食品知名度和市场占有率。 三是完善农业服务体系。加强农技、水利、农机和农村金融服务。特别注重粮食、食用菌、畜牧、渔业、林木、经济作物等良种的优选和优质基因的培育，并形成本土在良种方面有自主知识产权和核心竞争力的科研机构和龙头企业，提高精品农业可持续竞争力。建立市与县（市）区联动的农业担保体系，鼓励金融主体开展土地经营权、林权、宅基地及农村住宅抵押贷款等业务，全市农业保险面积发展到500万亩。 创新营销服务，充分利用电商平台等各种营销手段，让牡丹江特色农产品叫好又叫座。四是推进农业综合改革。出台农村产权交易管理办法，启动运行农村产权交易平台，完成土地承包经营权确权登记测量工作。

　　（四）大力发展现代服务业。加快旅游产业发展，贯彻市委加快旅游产业创新发展的实施意见，加速建设国际旅游目的地和全国重点旅游度假胜地。加强旅游产品开发，深度开发“湖雪林边俗红特”及火山、瀑布、湿地、文化、影视拍摄基地等丰富的旅游资源，实施旅游“强点拓线结网”工程，重点打造以镜泊湖为龙头的“江湖旅游走廊”和以冰雪为主题的“哈亚牡冰雪旅游之路”，建设有市场需求的“吃住行游购娱”项目，加大旅游纪念品开发和营销，打造中俄韩朝旅游商品集散地,全市旅游人数和旅游收入增长20%以上。提高旅游服务水平，积极引进世界高端酒店管理公司，加强旅游企业与高校旅游专业合作，提高在地旅行社服务水平和导游综合素质。加强旅游管理，争取省里支持，打破体制障碍，加强旅游市场质量和价格监管，提高国内外游客24小时投诉处置和应急响应能力。加大旅游合作，做好长白山管委会、哈尔滨旅游集团等合作项目；促进我市旅行社与国内外一流旅行社和航空公司合作。大力加强旅游营销，利用现代媒体及立体营销手段，叫响牡丹江的旅游品牌。加快商贸转型升级，大力发展电子商务等新兴业态，加快国家跨境电子商务服务试点建设，推进市经开区国家电子商务示范基地建设。发展壮大物流业，编制实施“物流体系专项规划”，合理布局全市生产性物流、生活性物流、过境物流，建立综合性物流信息服务及交易平台，提高服务水平，降低综合成本。推进省级冷链物流产业示范区建设。提升金融服务能力，强化金融生态环境建设，推动金融产品创新，拓展民间借贷登记中心功能， 新建村镇银行等金融机构3家以上。将上市融资作为企业做大做强的重要途径，用政策和产业基金引导激发企业上市的积极性，鼓励企业兼并重组，通过多种渠道走入资本市场。促进房地产健康发展，积极推进养老地产、医养结合地产、旅游地产、城市综合体建设，积极化解房地产库存。

　　（五）奋力发展更高层次的开放型经济。对外开放，特别是对俄对韩开放，是牡丹江最大的优势所在。一是构建开放型产业体系。发挥外贸龙头企业带动作用，在俄粮回运加工和俄林木、矿石、水产品、油气加工上形成产业规模，在轻工、机电、电子产品等方面培育出口龙头企业和产业群。支持境外园区发展，争取华宇经贸合作区晋升国家级。 推动外贸企业提档升级、做实做强，加快跨境电商发展。 抓好韩国产业园建设，在机器人、小电器、轻工产品上吸引韩国中小企业集群发展。 拓展与美日欧的产业合作， 推动高端装备、医疗仪器、现代农业、生物医药、新材料等产业加快发展。大力发展开放型农业，全市农产品出口基地达到37万亩，在俄农业合作开发面积达到500万亩。 做好开放政策谋划和项目策划，主动对口对接各相关部委，最大限度争取政策和项目，力争在中俄跨境游免签、自驾游、卢布人民币在域内自由兑换等政策上有所突破。二是加快基础设施互联互通。推进东宁口岸界河桥改造和牡符跨境高铁及东珲、东乌高等级公路等项目,对接“滨海1号”中俄大通道，推动“哈牡绥俄亚”大通道常态化运营。充分发挥绥芬河国家综合保税区的辐射带动作用，推进市经开区综合保税物流中心和东宁跨境经济合作区建设，开通牡丹江机场航空货运，力争华晟内陆港实现班轮制运输。三是扩大交流合作。深化与俄、韩等国家在教育、科技、文化、医疗、体育、旅游等领域的合作，开展中俄媒体交流年系列活动。四是优化开放环境。开通24小时俄语、韩语旅游服务热线，鼓励牡丹江中医院等医院开展针对俄罗斯公民的医疗服务。在主要交通干道、重点旅游景区、星级宾馆酒店等公共场所增加俄语和韩语标识，建设中俄小商品交易大市场。

　　（六）协力推进城乡一体化发展。按照市委“三个统筹”新要求，加速推进全国文明城市创建工作。坚持高水平规划，抓好城市总体规划修改、城郊乡镇总体规划、沿江两岸景观设计、城市交通改善以及海绵城市等专项规划编制，严控老城区商品房开发。 深化城市建设投资方式改革，引入PPP等投融资模式，多渠道筹措建设资金。打通城市中轴，建设南北太平路贯通下穿隧道、北太平路。高标准建设城市标志性建筑及桥梁，开工建设牡丹江高铁南站、虹云桥等工程，牡丹江大桥10月份竣工通车。加快江南新城建设，加速绿化亮化美化和新型商圈打造， 创建最优质的教育、医疗、体育、文化等公共服务。 提升城市品位和综合承载力，提升光华街、太平路、西三条路立面品位，加快推进公交走廊、地下管廊、“三供三治”等工程，实现城区供水管网全覆盖。 实施“五小工程”，建设小公园、小广场、小绿地、小市场、小停车场100个。提升城市管理水平和社会文明程度，打好市容管理、道路交通等城市环境“八个攻坚战”，开展主城区破旧建筑物拆除改造工程，确定一批“严管街”、“严管区”，整治不文明行为，解决市场脏乱差问题，力争城市面貌焕然一新。深入推进国家新型城镇化综合试点，加快新型城镇化和美丽乡村建设。统筹推进配套改革，推进温春国家建制镇示范试点。突出抓好G10、G11公路和铁路沿线及重点景区周边环境整治，新建美丽乡村示范村20个、达标村100个，改造农村泥草（危）房8000户。

　　（七）着力加强生态文明建设。全面启动国家生态文明先行示范区建设。加大环境污染治理力度。深入开展市区大气污染、污水排放、固体废物堆放、噪声等综合环境整治。推广使用清洁能源，严控秸秆焚烧，严控燃煤质量， 取缔黄标车6000辆、小锅炉65台，新增燃气入户2万户。启动实施工业污染源全面达标排放计划和近零碳排放区示范工程，开展电力、造纸、橡胶等行业除尘、脱硫、脱硝综合治理。探索环境第三方治理模式。开展绿色生活“十进”活动，引导社会公众选择绿色低碳消费模式和生活方式。创建更优生态环境。启动城区及周边裸露山体保护治理工程，恢复森林植被。推进封山育林和25度以上坡体耕地退耕还林，全市治理水土流失36万亩。加强牡丹江饮用水源地综合整治和海浪河源头生态保护，建设牡丹江沿江和海浪河2个国家湿地公园。启动“江湖连通净化工程”，加快市区湖泡治理。 开展“增绿护绿行动”，市区新增绿地30万平方米以上。畅通城区通风走廊，今后凡影响城区通风的建筑一律不予审批，让百姓看得见山、望得见水，把全市人民对天蓝水净山绿的美好期待变成现实。

　　（八）倾力提升文化软实力。一是强化文化引领。大力弘扬社会主义核心价值观、中华优秀传统文化、地域特色文化和以“创新、实干、开放、诚信”为主要内容的城市精神，筑牢百姓精神家园。深入实施市民文明素质提升行动计划，抓好道德建设“十项工程”。二是发展壮大文化产业。培育靺鞨绣、蚕翼绣、木制工艺品等本地民族民俗特色文化产业，培育动漫、创意、影视、广告、会展、演艺等时尚产业，注重文化融入“互联网＋”、文化融合旅游产业，鼓励中俄文化艺术品交易中心、横道影视城、文化创意产业园等文化产业载体发展，重视旅游景区的驻场演出。三是大力发展文化事业。启动渤海国上京龙泉府遗址列入世界遗产清单目录工作，推进中东铁路建筑群、牡丹江边墙等文化遗产保护与开发。实施文化惠民工程，完善四级公共文化服务网络。办好朝鲜民族艺术节。广泛开展全民健身活动，建设江南全民健身体育走廊，把市体育馆改建成全民健身中心。启动城市规划馆和博物馆前期工作。

　　（九）竭力保障和改善民生。 深入落实市委 “工程式推进民生工作”要求， 全力推进“九项民生工程”，务实办好30件利民实事。一是全面推进精准扶贫、精准脱贫。 落实打赢脱贫攻坚战行动计划， 遵循“六个精准”、“五个一批”的要求， 按照人均收入达到3300元、贫困人口不愁吃穿和义务教育、 基本医疗、住房安全有保障的标准，扎实推进脱贫攻坚工作， 确保12个建档立卡贫困村摘帽、1.3万农村人口实现脱贫。二是促进就业增收。 援助就业困难人员1.7万人次，新增城镇就业5.5万人， 城镇登记失业率控制在4.3%以内。确保城镇居民收入保持全省前列、农民收入保持全省首位、机关事业单位干部职工工资收入稳定增长，衔接好国家和省企业职工基础养老金统筹改革工作，完善离退休人员养老金正常增长机制。三是切实加强社会保障。五项社会保险新增参保人员5万人，提高城乡低保标准，全面实行大病保险制度。 建立救急难“四级联网”机制，加强重特大疾病医疗救助，全面实施临时救助制度，编实织密困难群众基本生活安全网。 四是推进保障性住房建设。改造棚户区6000户，安置回迁居民1.6万户。进一步加强物业管理，确保无弃管小区。五是加快发展社会事业。促进义务教育均衡发展，鼓励发展普惠性幼儿园，推进普及高中阶段教育，加快发展现代职业教育，完成80所农村薄弱学校改造。筹集助学资金2000万元，资助家庭经济困难学生1.1万名，实现家庭经济困难学生资助体系全覆盖。 支持牡丹江大学向本科办学质量看齐。支持在牡高校发展，加强校地全面合作。深入落实“健康牡丹江”行动计划， 加快推进城乡三级卫生服务网络建设， 新型农村合作医疗覆盖率、参合率和大病保险制度开展率均达到100%。 积极落实“全面二孩”政策。 重视发展养老事业和残疾人事业，鼓励医养结合，鼓励社会力量兴办养老事业，新增养老床位1000张，完善“空巢老人”、“失独家庭”等关爱服务体系，建设残障人员综合服务中心和10个“留守流动儿童家园”。六是切实维护平安稳定。深入贯彻落实新安全生产法和条例，常态化开展安全生产大检查，完善突发事件监测预警体系和应急响应机制，进一步加大小煤矿关闭力度，坚决防范遏制重特大事故。争创全省食品安全城市。推进社会治理体系深度改革，做好社区居委会换届选举工作。深入推进信访工作制度改革，解决好房屋办照、居民回迁、一房多售、农民工工资拖欠等遗留问题。全面加强立体化社会治安防控体系建设，持续开展平安雪城、春季护航、百日护游专项行动，严防严打各类刑事犯罪，争创“全国平安市”。更加注重民族团结和宗教和谐，争创“全国民族团结进步示范市”。

　　四、加强政府自身建设

　　　　深入贯彻国家和省推进政府治理能力现代化战略部署。 一是加快学习型政府建设。 践行“五大发展理念”，深学透悟笃行习近平总书记系列重要讲话精神，紧紧把握党中央国务院、省委省政府、市委精神，紧紧盯住市场趋势、政策出台动向、科技前沿动态、资本投向， 紧紧抓牢重大政策落实和重大项目、 重大工程、重大平台建设， 不断提高政治理论水平、驾驭市场能力、掌控社会本领、 服务百姓实力、科技创新活力，筑牢防腐拒腐根基。二是加快法治政府建设。严格执行政府重大决策法定程序，强化政府权力运行制约和监督，全面推行政府法律顾问制度。严格权力清单、责任清单管理，严抓责任追究。深入推进行政综合执法改革，加大城市管理、资源管理、市场秩序、环境保护等方面的综合执法力度，抓好文化旅游领域国家试点综合执法改革。配合市人大做好地方立法权争取工作，建立健全我市行政法规体系，力争成为全国地级市法规体系建设与创新的标准。全面启动“七五”普法，加强政府工作人员的学法、用法、守法意识和行为规范，推进政府治理法治化。三是加快创新政府建设。深化政府管理体制改革，围绕服务自然人、法人“两个人”，抓住经济、社会两条线，集成政府职能，构建服务平台。围绕构建法人的经济服务平台，集成经济部门，加强要素服务、市场服务、产业链服务、园区载体服务，形成对企业服务和市场监管的合力。围绕构建自然人的生活服务平台，集成社会服务部门，联合社会、市场力量，对自然人从生老病死、业学行住、急难险弱等方面进行全方位联动服务，让百姓更加方便快捷。加大简政放权力度，能够取消的行政审批事项全部取消，各种不合理收费全部清理；推行“两随机一公开”政府监管模式；推进市、区两级政府事权改革，权力下放、重心下移；市镜管委、经开区和阳明区管理体制改革要取得实效；推进公共资源市场化改革。四是加快服务型政府建设。推进网上办理、后台处理、上门服务，一般项目和鼓励类重点项目建设审批时限减至45个和30个工作日，工业项目建设审批时限减至15个工作日；严格执行企业“门禁制度”和涉企收费“清单制”，全面清理规范中介机构收费标准； 推进“一屏妥”政府网上服务，打造与百姓和企业互动的4.0版政府在线服务。推进数字化政府建设，集成政府信息管理平台；构筑卫星遥测遥感、无人机巡逻、固定和移动视频监控、百姓手机举报相结合的全方位可视化城市监控的“天罗地网”。探索执法监管分离，防止监而不管、权力寻租。用现代管理理念、现代技术手段、法治精神，实现城市管理标准化、法规化、精细化、可视化、网络化。五是加快廉洁政府建设。巩固党的群众路线教育实践活动和“三严三实”专题教育成果，严格执行《中国共产党廉洁自律准则》和《中国共产党纪律处分条例》。牢固树立过紧日子思想，严控“三公”经费。全力优化经济发展环境，加强企业投诉中心建设，着力解决不作为、慢作为、乱作为等扰乱发展环境问题。加强作风建设，落实省委“四转”要求，强化敢于担当、善于担当的责任意识；保持事不过夜、事必躬亲、事无巨细的工作状态；提升科学理性、依法行政、驾驭市场的专业水准；形成“深细严实勤俭廉”的工作作风，打造一支政治可靠、业务精湛、作风过硬、人民满意的政府干部队伍。

　　自觉接受人大法律监督、政协民主监督和社会舆论监督，高质量、高效率办好提案议案。

　　各位代表， 让我们在市委正确领导下，牢记人民重托， 肩负时代使命，勇于担当、攻坚克难，真抓实干、只争朝夕，为牡丹江夺取全面建成小康社会决胜阶段新胜利而努力奋斗！
